# Exploring the interaction between T-cell antigen receptor-related genes and MAPT or ACHE using integrated bioinformatics analysis

**DOI:** 10.3389/fneur.2023.1129470

**Published:** 2023-03-28

**Authors:** Wenbo Guo, Xun Gou, Lei Yu, Qi Zhang, Ping Yang, Minghui Pang, Xinping Pang, Chaoyang Pang, Yanyu Wei, XiaoYu Zhang

**Affiliations:** ^1^College of Computer Science, Sichuan Normal University, Chengdu, China; ^2^College of Life Science, Sichuan Normal University, Chengdu, China; ^3^College of Mathematics and Physics, Chengdu University of Technology, Chengdu, China; ^4^West China School of Basic Medical Sciences and Forensic Medicine, Sichuan University, Chengdu, China; ^5^National Key Laboratory of Science and Technology on Vacuum Electronics, School of Electronic Science and Engineering, University of Electronic Science and Technology of China, Chengdu, China

**Keywords:** Alzheimer's disease, RNA sequencing, neuroinflammation, random forest classifier, ensemble learning, T-cell antigen receptor, eigenvalue decomposition, protein docking

## Abstract

Alzheimer's disease (AD) is a neurodegenerative disease that primarily occurs in elderly individuals with cognitive impairment. Although extracellular β-amyloid (Aβ) accumulation and tau protein hyperphosphorylation are considered to be leading causes of AD, the molecular mechanism of AD remains unknown. Therefore, in this study, we aimed to explore potential biomarkers of AD. Next-generation sequencing (NGS) datasets, GSE173955 and GSE203206, were collected from the Gene Expression Omnibus (GEO) database. Analysis of differentially expressed genes (DEGs), gene ontology (GO) functional enrichment, Kyoto Encyclopedia of Genes and Genomes (KEGG) pathway enrichment, and protein-protein networks were performed to identify genes that are potentially associated with AD. Analysis of the DEG based protein-protein interaction (PPI) network using Cytoscape indicated that neuroinflammation and T-cell antigen receptor (TCR)-associated genes (*LCK, ZAP70*, and *CD44*) were the top three hub genes. Next, we validated these three hub genes in the AD database and utilized two machine learning models from different AD datasets (GSE15222) to observe their general relationship with AD. Analysis using the random forest classifier indicated that accuracy (78%) observed using the top three genes as inputs differed only slightly from that (84%) observed using all genes as inputs. Furthermore, another data set, GSE97760, which was analyzed using our novel eigenvalue decomposition method, indicated that the top three hub genes may be involved in tauopathies associated with AD, rather than Aβ pathology. In addition, protein-protein docking simulation revealed that the top hub genes could form stable binding sites with acetylcholinesterase (ACHE). This suggests a potential interaction between hub genes and ACHE, which plays an essential role in the development of anti-AD drug design. Overall, the findings of this study, which systematically analyzed several AD datasets, illustrated that LCK, ZAP70, and CD44 may be used as AD biomarkers. We also established a robust prediction model for classifying patients with AD.

## 1. Introduction

Alzheimer's disease (AD), which is the most common form of dementia in the elderly, is regarded as a central nervous system disorder (1). Considerably more people over the age of 60 years live with disabilities associated with AD (11.2%) than those associated with stroke (9.5%) or musculoskeletal disorders (8.9%) (2). After the age of 65 years, the probability of developing AD doubles every 5 years, thereby affecting 30–50% of people over the age of 85 years (3). Although the pathogenesis of AD remains largely unknown, the deposition of amyloid- β (Aβ), formation of neurofibrillary tangles (NFTs) due to MAPT hyperphosphorylation, and neuroinflammation are considered to be the leading causes (4–6). Moreover, rapid advances in AD research have resulted in numerous different clinical drugs being developed. Currently available drugs, such as inhibitors of acetylcholinesterase (ACHE) and butyrylcholinesterase (BCHE), are able to alleviate cognitive and memory decline linked to AD (7–9). Despite the availability of drugs for delaying the progress of Alzheimer's Disease (AD) at the clinical level, there is currently no cure for the disease (10, 11). The absence of dependable biomarkers for early diagnosis and drug design presents a significant challenge to AD research. Therefore, it is imperative to investigate the underlying pathological mechanisms of AD and explore potential biomarkers for the disease.

Over the past few decades, next-generation sequencing (NGS) has been extensively used to study the pathological mechanisms underlying AD (12). Re-analysis of the vast amounts of sequencing data produced by NGS experiments may help understand the mechanisms underlying AD progression and develop drugs designed to treat this disease effectively. Numerous studies have been conducted on NGS public sequencing data of AD. Himanshu Narayan Singh used NGS bioinformatics analysis to determine that *DYNLL1* and *KLRN* were significantly associated with AD, which suggested that these proteins may constitute the genetic basis of AD (13). Furthermore, gene ontology (GO) and Kyoto Encyclopedia of Genes and Genomes (KEGG) analyses performed by Fang indicated that certain functions, such as taxis and cell-cell signaling, were strongly associated with AD (14). Although numerous studies have investigated the differential expression of AD genes, the results of only some have been verified *via* mathematical models or biological experiments. Thus, in this study, we designed two efficient classification models that predict patients with AD based on the expression levels of differentially expressed genes (DEGs).

In this study, we aimed to identify DEGs between AD and non-AD individuals in two different datasets. Next, the processes and pathways enriched by these DEGs were elucidated, and CytoHubba was utilized to identify the top hub genes among all DEGs, resulting in the identification of neuroinflammation and T-cell antigen receptor (TCR)-related genes as top hub genes. Subsequently, we developed a novel correlation analysis based on eigenvalue decomposition methods. This new method was used to analyse assortativity between the top hub genes as well as identify the primary hallmark genes of AD to determine which pathology was more critical. In addition, another data set was used to develop two machine learning models that disclose patients according to the expression levels of hub genes. Finally, we further investigated the potential effects exerted by the top hub genes on drug designs involving ACHE or BCHE inhibitors. In this study, we aimed at providing valuable insights into the mechanisms underlying neuroinflammation and the functioning of TCR-related genes in AD.

## 2. Materials and methods

### 2.1. Datasets information

Two publicly available high-throughput RNA sequencing datasets, GSE173955 (GPL18460) (15) and GSE203206 (GPL20301) (16), were extracted from the Gene Expression Omnibus (GEO; https://www.ncbi.nlm.nih.gov/geo/). In the GSE173955 dataset, the AD group consisted of eight biological replicates, whereas the normal group consisted of 10 biological replicates, and all samples were repeated once for the purpose of technical duplication, which effectively minimized sequencing error (17). It used hippocampal tissues from eight AD and ten control (non-AD) autopsy samples of Hisayama residents, Japan. The assessment of AD pathology was conducted according to the Consortium to Establish a Registry for Alzheimer's Disease (CERAD) guidelines and the Braak stage. The age range of the samples was 55–100 years old. Moreover, the GSE203206 dataset used brain tissue samples obtained from the Brodmann Area 17 (Bm-17) of the occipital lobe (OL) of 40 AD patients and 8 healthy, non-demented control (NDC) samples preserved at the UC San Diego Shiley-Marcos Alzheimer's Disease Research Center. The AD samples were selected based on their lack of alternative diagnosis, APOE status, and age at onset (AAO). Three cognitive evaluation scores were used to classify the selected patients as AD or NDC. Each brain sample was staged based on the concentration of Neurofibrillary Tangles (NFTs) in different brain regions. We also utilized two additional datasets, GSE15222 and GSE97760, to validate the performance of our machine learning model and feature analysis results, with the aim of examining the generalizability of our findings across different datasets (18). Table 1 provides a comprehensive overview of the datasets utilized in this study.

**Table 1:** The description of different datasets.

**Dataset**	**Tissue**	**AD definition**	**Demographics**
GSE173955	Hippocampal	CERAD and Braak stage	Japan; Age 55–100
GSE203206	Occipital lobe	Cognitive scores and NFT staging	United States; Age 41–97
GSE15222	Temporal cortex	CERAD and Braak stage	European
GSE97760	Blood	Cognitive scores	United States; All female; Age 59–91

We selected the datasets as following criteria: (1) The datasets should published in one years. (2) The datasets should contain only AD and NON-AD groups. (3) They should be RNA NGS data, since it represents the active genes that are being transcribed and translated to produce proteins, and changes in RNA expression levels can reflect changes in cellular function and physiology. (4) The datasets should include at least thirty replicates. AD is a complex disease that involves multiple genetic and environmental factors, and a small replicates may not adequately represent the heterogeneity of the disease or provide enough statistical power to identify significant biomarkers. Additionally, a larger replicates can improve the generalizability and reproducibility of the findings, allowing for more robust conclusions and potential translation into clinical practice.

### 2.2. Pre-processing of datasets

As GSE173955 only provided fasta files, the raw fasta files of GSE173955 need to be converted into gene expression values in this study. Firstly, the raw data was downloaded using the “Aspera” tool due to its fast transfer speeds and error detection and correction mechanisms. Secondly, "Fastp" software was used to trim and filter reads, where reads with lengths below 50 bp, complexities below 30%, or mean quality scores below 20 were cut (19). Thirdly, “Hisat2” was used to map the nucleotide sequences to their corresponding genes using the Hg19 reference genome available on Ensembl. Fourthly, “StringTie” was used to assemble the transcripts, estimate their abundances, and output the results in a gene expression matrix. Lastly, we used the Voom approach with TMM normalization in this article (20). This method corrects for technical variability in RNA-seq data caused by sequencing depth, library composition, and other sources of variation. TMM normalization ensures that read counts are comparable between samples, while the Voom transformation accounts for the mean-variance relationship. Voom is statistically more robust when library sizes vary greatly, as in GSE173955. The article provides code for each step and highlights the importance of each tool and the reasoning behind the parameters used in each step in Supplementary section 1.

### 2.3. Identification of DEGs

In our study, we performed differential expression analysis using the "limma" package (version 3.48.3) of R language [R version 4.1.0 (2021-05-18)], following the instructions provided in the Limma manual (21). To account for potential confounding variables, we created a four-column design matrix that included disease status, age, and gender as covariates (Supplementary Table 1). We used a linear model to fit the normalized data to this design matrix, utilizing the “lmFit” function provided by Limma. By incorporating these covariates into the model, we were able to adjust for their effects on the gene expression data.After fitting the model, we calculated the empirical Bayes moderated *t*-statistics and *p*-values for each gene using the “eBayes” function in Limma. To account for multiple testing, we adjusted the *p*-values using the Benjamini-Hochberg procedure method. Specifically, we used the “topTable” function in Limma to generate a table of differentially expressed genes, sorted by their adjusted *p*-values (22).

The thresholds for identifying differentially expressed genes in our study were set at |*logFC*| > 1.5 and ρ < 0.05. These criteria were chosen based on previous studies and our own preliminary analyses, and allowed us to identify a set of genes that were significantly differentially expressed between the two groups being compared. Next, “ggplot2” was used to visualize all up-regulated and down- regulated DEGs *via* a volcano plot, while the package "pheatmap" was employed to display the correlation between DEGs and samples *via* a heatmap.

### 2.4. Enrichment analysis

GO analysis is widely used to describe the biological attributes of genes and gene products associated with specific biological processes (BPs), molecular functions (MFs), and cellular components (CC) (23). BPs involve a wide range of processes, which can be described by an ordered combination of molecular functions. MFs are used to annotate the molecular level functions of genes or gene product, whereas CCs are utilized to elucidate the locations and structures of genes. KEGG enrichment analysis is used to annotate genomic and chemical information to particular pathways (24). We used "clusterProfiler," an R package, to perform KEGG and GO pathway analysis for identifying biological pathways and functional categories that are enriched with differentially expressed genes (DEGs) between Alzheimer's disease (AD) and non-AD groups. The enrichment analysis was performed separately for upregulated and downregulated genes. The maximum *p*-value was set at 0.05 and the maximum q-value (adjusted *p*-value) was set at 0.2, indicating that any pathways beyond these values were considered non-significant. This approach enabled the identification of key biological pathways and processes that are involved in AD pathogenesis and provided insight into the underlying mechanisms of this complex disease.

### 2.5. Protein-protein interaction (PPI) and hub genes analysis

To investigate the interaction between differentially expressed genes (DEGs), we constructed a protein-protein interaction (PPI) network using the Search Tool for the Retrieval of Interacting Genes (STRING; https://string-db.org/cgi/network.pl). To ensure the reliability of the interactions, a minimum interaction score of 0.99 (default: 0.50) was set for PPI analysis, and the option to 'hide disconnected nodes in the network' was enabled to filter out networks with an excessive number of genes. To further analyze the network, we utilized the CytoHubba plugins in Cytoscape, which rank nodes and identify hub genes based on the degree weight (25).

### 2.6. Correlation analysis of top hub genes

The AD database (http://www.alzdata.org/) was used to examine the expression levels of hub genes, relationship with the AD PPI network, and pathology of tau or abeta (26). This online database uses convergent functional genomic (CFG) analyses of gene profiles in AD and non-AD groups from various GEO datasets. The CFG method provides extra information by integrating AD- related evidence with DNA variations linked to disease susceptibility, the PPI network involving APP, PSEN1, PSEN2, APOE, and MAPT, and predictive scores obtained from mouse AD models.

### 2.7. Construction of the random forest and ensemble prediction model

Random forest, proposed by Breiman, is a state-of-the-art learning algorithm which performs classifications based on decision trees (27). It can predict whether a sample has AD based on gene expression levels. In this study, two random forest models were constructed using the input of all genes and hub genes. The difference in prediction accuracy indicates the importance of hub genes in AD development. Ensemble learning is considered a variable solution for prediction. It trains and combines different machine learning predictions to improve the predictive performance of a single model (28). After constructing the single random forest model, an ensemble prediction model, consisting of a random forest binary classifier (RF), Gaussian mixture model (GMM), linear model (LM), and support vector machine binary classifier (SVM), was established to predict AD patients *via* hub gene expression. Another dataset, GSE15222, consisting of 187 controls and 176 AD cases, was used to train and test machine learning models for the purpose of validating the correlation between hub genes and AD in different datasets (18).

All models were constructed and validated *via* the sklearn package of Python3.7 (29), using 80% and 20% of GSE15222 data as training and as testing data, respectively. The random forest model was developed using the Gini criterion, wherein the radial basis function acts as the kernel of SVM (30). Changing the voting standardization of the ensemble model from hard to soft, conferred an outstanding capability for predicting diabetes mellitus (31) and cardiovascular events, such as chronic thromboembolic pulmonary hypertension (cteph) (32). Based on the knowledge of domains, the weights of the model were set at 2 for random forest and SVM, and 1 for GMM and LM.

### 2.8. Visualization of machine learning results

A receiver operating characteristic (ROC) curve was plotted to evaluate the performance of the classifier models. This is a widely used graphical representation which demonstrates the performance of a binary model (33). The area under the receiver operating characteristic curve (AUC) was calculated to evaluate classification accuracy. The x-axis and y-axis of the ROC represent false positive and true positive rates, respectively. Furthermore, a nomogram figure was used to estimate the probability of AD using a single numerical score. This is a user-friendly graphical interface for clinical encounters (34). All figures were plotted using the "matplotlib.pyplot" package of python if not specified.

### 2.9. Eigenvalue analysis

Correlation analysis revealed the coordination between genes and diseases. However, a coordination between multiple genes was not observed. Therefore, we utilized a novel method for analysing coordination between several genes based on the eigenvalue decomposition method. Let $X_{con}={\left\{{\vec{x}}_{1}^{con},{\vec{x}}_{2}^{con},...,{\vec{x}}_{n}^{con}\right\}}^{t}$ and $x_{ad}={\left\{{\vec{x}}_{1}^{ad},{\vec{x}}_{2}^{ad},...,{\vec{x}}_{n}^{ad}\right\}}^{t}$ denote the matrix of hub gene expression values after Z-score standardization of the normal and AD groups, respectively, and *n* denote the number of hub genes. We then constructed two groups of inner product matrices, *R*_*con*_ and *R*_*ad*_, consisting of vectors *a*_*i*_ and *b*_*i*_, respectively. Below is an example of *R*_*con*_ construction.


$${\vec{a}}_{i}^{=}\left(x_{i1},x_{i2},...,x_{im}\right)$$

$${\vec{a}}_{i}\cdot {\vec{a}}_{j}=x_{i1}x_{j1}+x_{i2}x_{j2}+...+x_{im}x_{jm}$$

$$R_{con}=\left[\begin{array}{c} {\vec{a}}_{1}\\ \vdots \\ {\vec{a}}_{n} \end{array}\right]{\left[\begin{array}{c} {\vec{a}}_{1}\\ \vdots \\ {\vec{a}}_{n} \end{array}\right]}^{T}=\left[\begin{array}{ccc} {\vec{a}}_{1}\cdot {\vec{a}}_{1} & \cdots & {\vec{a}}_{1}\cdot {\vec{a}}_{n}\\ \vdots & \ddots & \cdots \\ {\vec{a}}_{n}\cdot {\vec{a}}_{1} & \cdots & {\vec{a}}_{n}\cdot {\vec{a}}_{n} \end{array}\right]$$

where *m* is the number of samples, *n* is the number of hub genes, and *i* and *j* represent the *i* − *th* and *j* − *th* hub genes in *X*_*con*_. *R*_*con*_ and *R*_*ad*_ are correlation matrices constructed using the correlation coefficient (inner product), which demonstrates the relationship between the hub genes. However, valuable information, such as the whole coordination between genes and diseases, is hidden in the matrix. The eigenvalue decomposition method can reveal useful information as follows:


$$R_{con}=Q\left[\begin{array}{ccc} \lambda & \; & \;\\ \; & \ddots & \;\\ \; & \; & \lambda_{i} \end{array}\right]Q^{-}1$$

where λ, the eigenvalue of the semi-definite matrix *R*, represents the eigen information on hub genes. It is important for *Q* to be an invertible matrix, guaranteeing that eigenvalue decomposition does not affect gene correlations. To compare λ in different groups, we transformed it into a percentage using the formula, $\lambda_{i}=\lambda_{i}/\overset{n}{\underset{k=1}{\sum }}\lambda_{k}$. The sum of λ should then be equal to 1, and the k = 1 value of each λ should be larger than 0 and smaller than 1. Moreover, another dataset, GSE97760 (10 AD samples and 9 non-AD samples), was used in this analysis to validate the results in different datasets.

This novel method can reveal the intrinsic characteristics and coordination between all genes. In this study, we combined the expression values of hub genes and *MAPT* and *APP*. Eigenvalue analysis was performed to compare the coordination between tauopathies and Aβ pathology.

### 2.10. Protein docking to explore the interactions between hub gens and choline

Choline, which is the most critical therapeutic drug used against AD, alleviates cognitive decline and accelerates the recovery of consciousness (35–37). Protein- protein docking simulation methods play a vital role in drug design. We used the high ambiguity riven protein to protein docking (HADDOCK) algorithm to predict the binding sites between hub genes and choline (38). The protein structural file in protein data bank (PDB) format was downloaded from the Alphafold2 database (39, 40). Discovery studio 2019 was used to remove water molecules from the proteins. The hub proteins of the above analysis were input as receptor proteins, and the two kinds of choline proteins, ACHE and BCHE, were used as the ligand and protein, respectively. The HADDOCK module was then run to predict the docking site and calculate the docking score. The results of the predicted model are shown in a Ramachandran plot (41).

## 3. Results

### 3.1. GSE173955 filtering and mapping

The Supplementary Table 2 represents the results of a sequencing experiment where raw data reads, clean data reads, and the quality of the clean data were measured for 10 different samples and 20 replicates in the GSE173955 dataset. The samples are denoted by their name, which can be divided into two groups—AD (Alzheimer's Disease) and CON (Control). The raw data reads represent the total number of reads obtained from the sequencing experiment. In the GSE173955 dataset, a total of 1,353,805,542 raw reads were generated from 40 samples, including 583,925,188 from Alzheimer samples and 769,880,354 from control samples. After removing adapters, short reads, low-quality reads, and bases, 1,307,630,392 clean reads remained, amounting to an average of 32,690,759 reads per sample. The clean data q30 rate represents the percentage of reads with a Phred quality score of 30 or higher, which indicates that the base call accuracy is 99.9% or higher. The q30 rate is an important quality metric in sequencing experiments, as higher accuracy reads result in more accurate downstream analysis. The q30 rate in GSE173955 datsets is above 94% for all samples, indicating high-quality data was obtained from the sequencing experiment. During the mapping process, 85.07% of the clean reads were successfully mapped to Hg19. The high mapping rate indicates that the sequencing data was of good quality and can be used for downstream analysis.

### 3.2. Identification of DEGs

**Figure 1:**
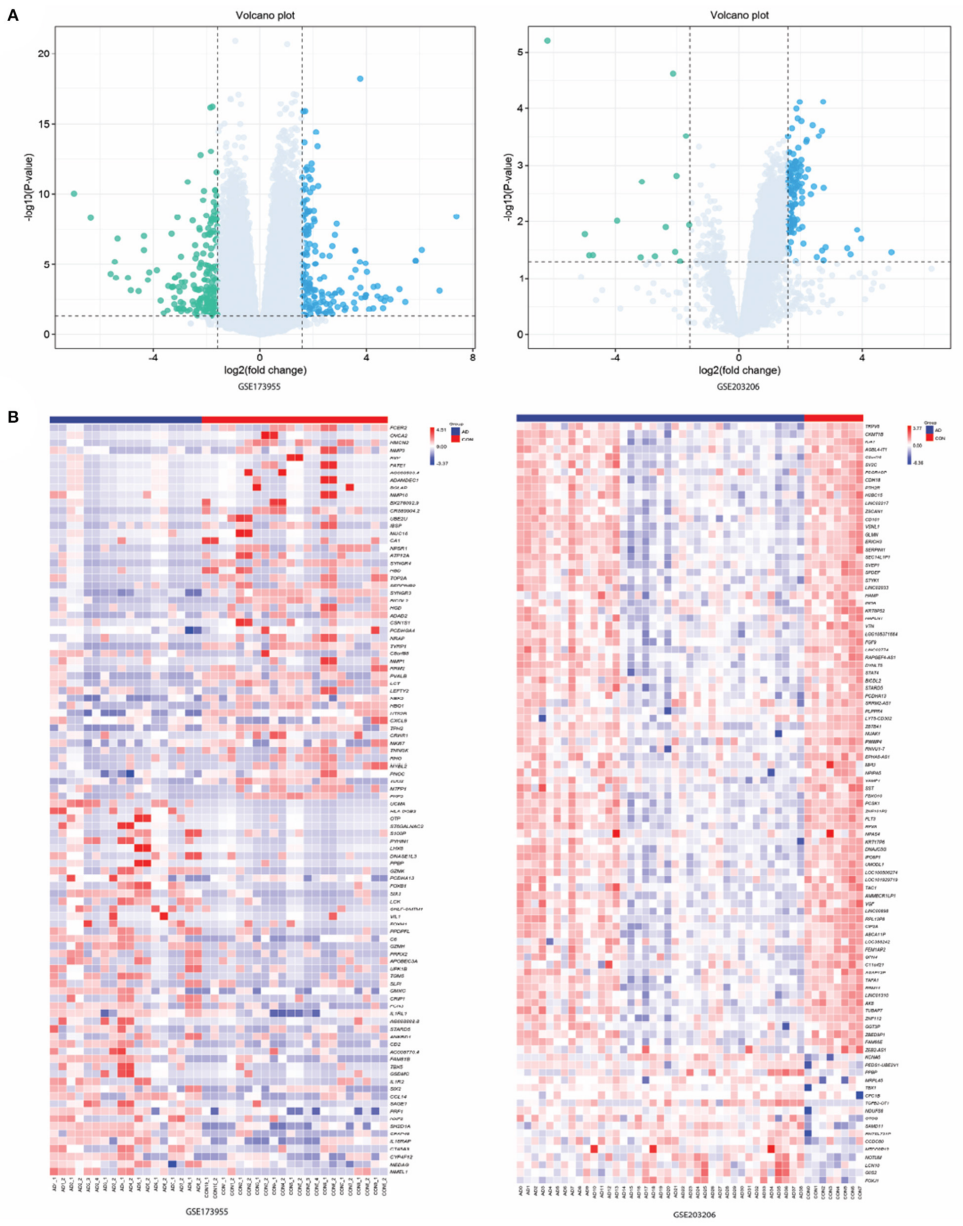
Results of screening differentially expressed genes (DEGs). **(A)** Volcano maps of gene expression in GSE173955 and GSE203206. Green data dots represent down-regulated DEGs. Data points in light blue and gray represent genes with up-regulated and not significantly different expressions, respectively. **(B)** Heatmap of top 100 DEGs in GSE173955 and GSE203206.

In this study, we utilized the "limma" package in R language to perform differential expression analysis. Specifically, we used the "voom" function to transform the Trimmed Mean of M (TMM) values, which is a recommended normalization method for RNA-seq data (20). Moreover, we also use the design matrix to adjust the gene expression data in our study. By incorporating these covariates into the linear model, we were able to control for their effects on the gene expression data and ensure that any observed differences in gene expression were not due to these factors. We identified a total of 448 DEGs in the GSE173955 dataset and 199 DEGs in the GSE203206 dataset. Among them, 211 and 182 genes were up-regulated, while 237 and 17 genes were down-regulated in the GSE173955 and GSE203206 datasets, respectively. To visualize the DEGs, we used a volcano plot, generated using the "ggplot2" package in R, to display all up-regulated and down-regulated DEGs in the datasets (Figure 1A). The volcano plot allowed us to visualize the relationship between the statistical significance and fold change of the DEGs, and to identify the most significant DEGs. We also generated heat maps of the top 100 DEGs, ranked by their adjusted p-values, using the "pheatmap" package in R (Figure 1B). These heat maps allowed us to visualize the expression patterns of the top DEGs across different samples and to compare the expression levels between different groups. Overall, our analysis identified a set of DEGs that were significantly differentially expressed between the two groups being compared.

### 3.3. Enrichment analysis of the DEGs

In this study, GO and KEGG enrichment analysis were performed to identify biological pathways and functional categories that are enriched with differentially expressed genes (DEGs) between Alzheimer's disease (AD) and non-AD groups. The analysis was performed separately for upregulated and downregulated genes. A total of 374 upregulated genes and 249 downregulated genes were identified and used in the enrichment analysis.

**Figure 2:**
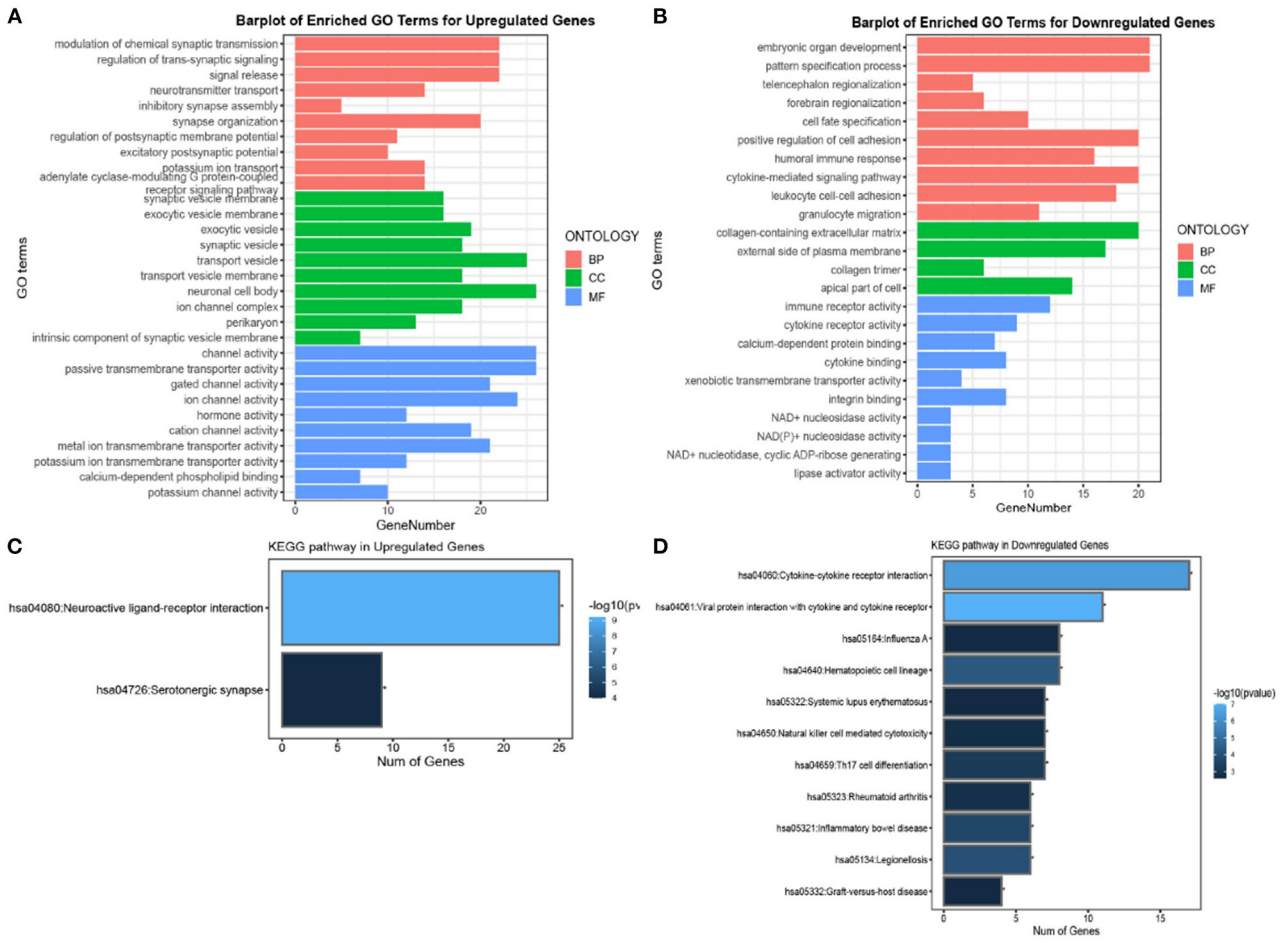
Gene ontology (GO) and Kyoto Encyclopedia of Genes and Genomes (KEGG) enrichment analysis of DEGs in AD. **(A, B)** Top 10 GO results in terms of molecular function, cellular component, and biological process category for upregulated and downregulated genes, respectively. **(C, D)** The significant KEGG pathways for upregulated and downregulated genes, respectively. The length and colors represent the number of genes and the log10 *P*-values.

For the GO analysis of upregulated genes, biological processes were primarily associated with signal release related functions, cellular components were primarily associated with "neuronal cell body," and molecular functions were dominated by "channel activity" and "passive transmembrane activity" (Figure 2A). In contrast, GO analysis of downregulated genes showed that biological processes were primarily associated with cell adhesion and signaling-related functions, cellular components were primarily associated with the "collagen-containing extracellular matrix," and molecular functions were dominated by immune and cytokine receptors (Figure 2B). For KEGG analysis of upregulated genes, 25 genes were significantly expressed in the neuroactive ligand-receptor interaction pathway, whereas the most significantly enriched pathway in downregulated genes was the cytokine-cytokine receptor interaction pathway, which involved 18 genes (Figures 2C, D). These results suggest that the pathogenesis of Alzheimer's disease involves complex molecular mechanisms that affect a range of biological processes, including signal release, cell adhesion, and immune response.

### 3.4. Hub genes analysis

**Figure 3:**
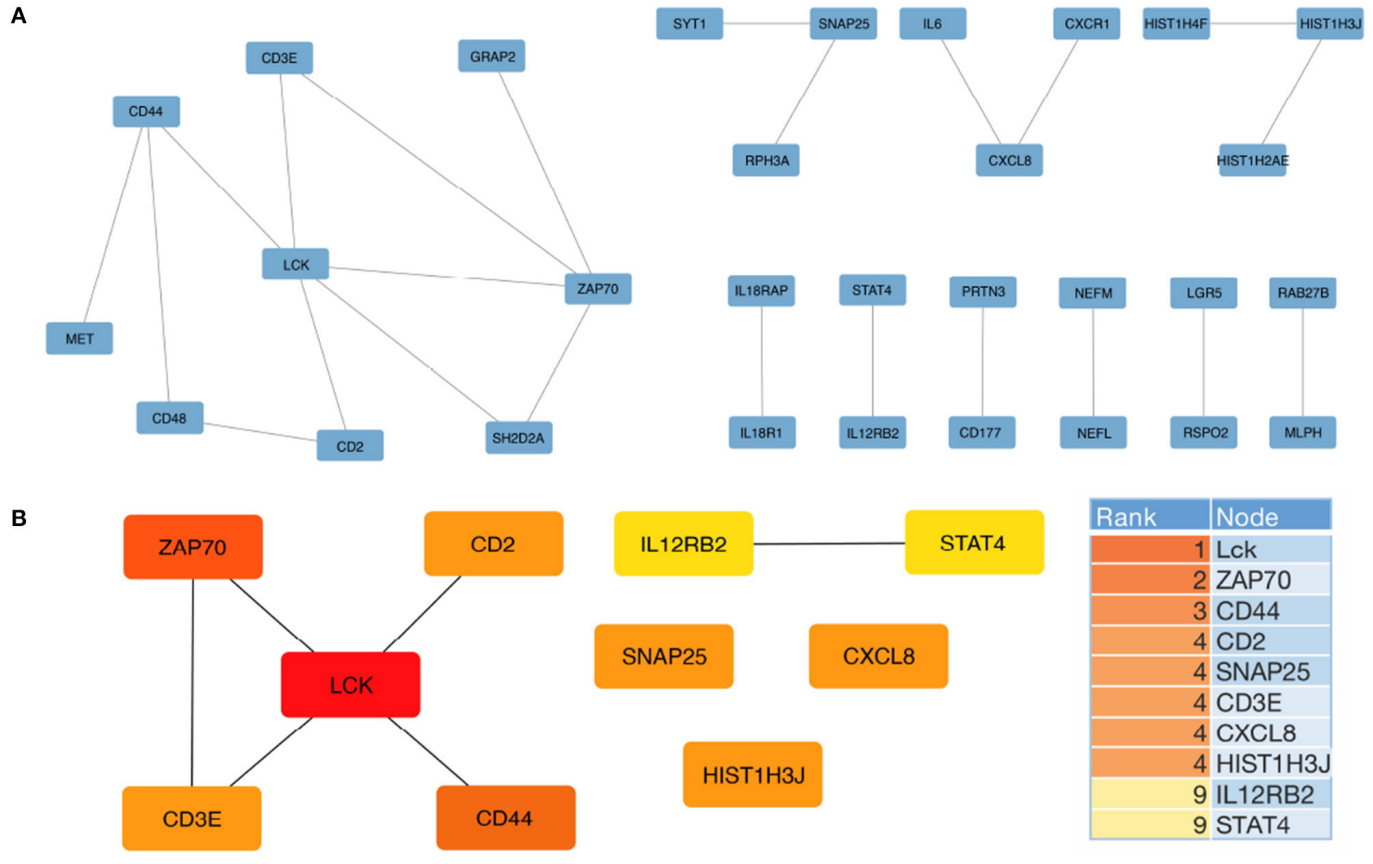
Protein-protein interaction (PPI) network for DEGs and hub gene identification was constructed using STRING and Cytoscape, respectively. **(A)** The PPI network of all DEGs was constructed using the STRING database with the minimum required interaction score set at 0.99. **(B)** The top 10 hub genes were analyzed using the CytoHubba plugin. The descending color from dark orange to yellow represents decreasing interaction intensity between genes.

Our study aimed to identify hub genes associated with Alzheimer's disease (AD) and evaluate their potential as biomarkers for the disease. To achieve this, we constructed a protein-protein interaction (PPI) network using the differentially expressed genes (DEGs) obtained from the Gene Expression Omnibus (GEO) dataset GSE15222. After excluding isolated nodes, we obtained a final PPI network consisting of 30 nodes and 22 edges (Figure 3A). Using the Cytoscape plugin CytoHubba, we identified the top ten hub genes, namely *Lck, Zap70, CD44, CD2, SNAP25, CD3E, CXCL8, HIST1H3J, IL12RB2*, and *STAT4* (Figure 3B). We further analyzed the top three hub genes, which enriched the same PPI network and were correlated with T cell activation, leukocyte cell-cell adhesion, and positive regulation of cell adhesion biological processes (Supplementary Table 3).

To verify the reliability of the identified hub genes, we checked their association with AD in the Alzheimer's database. Table 2 shown that five out of the top ten hub genes, namely *LCK, ZAP70, CD44, SNAP25*, and *IL12RB2*, were associated with the Alzheimer's pathological pathway (APOE, PSEN1, MAPT), and *LCK, ZAP70, CD44*, and *CD3E* were significantly differentially expressed in AD abeta and tau mouse models, respectively. This finding suggests that these hub genes may be important in the development and progression of AD and could potentially serve as biomarkers for the disease.

**Table 2:** The convergent functional genomic (CFG) result of hub genes.

**Gene**	**PPI^a^**	**Pathology cor abeta or tau^b^**	**CFG^c^**
LCK	APOE	*	4
ZAP70	APP,PSEN1,MAPT	*	3
CD44	APP,PSEN1	***	3
CD2	–	NA	1
CD3E	–	**	2
CXCL8	–	NA	0
SNAP25	MAPT	NA	2
HIST1H3J	–	NA	2
IL12RB2	APP,PSEN1,APOE	NA	2
STAT4	–	NA	1

a PPI: Target gene is significant correlate with APP, PSEN1, PSEN2, APOE, or MAPT (*P*-val < 0.05).

b Pathology cor abeta or tau: Correlation of target gene expression with AD pathology in abeta or tau in AD mouse models (NA: *P*-val > 0.05; *P-val < 0.05; **P-val < 0.01; ***P-val < 0.001).

c CFG: Total CFG score of target gene,range from 0 to 5.

**Figure 4:**
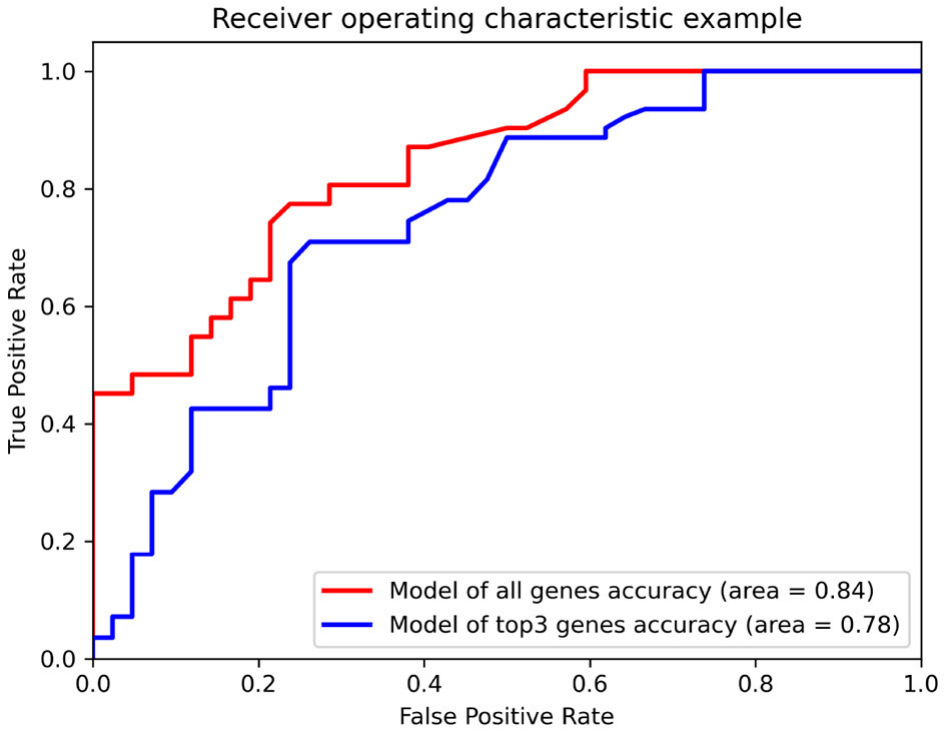
The receiver operating characteristics (ROC) of the random forest classifier model for the dataset, GSE15222. The red line and the blue line represent the model with all genes and top three hub genes as input, respectively. The area below the line represents the accuracy of the model.

Additionally, we evaluated the potential of hub genes as biomarkers using a random forest classifier model. The model was trained and tested using the GSE15222 dataset to verify its universality. The results showed that the model with all genes had an accuracy of 0.84 for predicting AD, whereas the model with only the top three genes had an accuracy of 0.78 (Figure 4). The area under the curve (AUC) for all predictive models was high, indicating that hub genes could be verified *via* the Alzheimer's database and mapping relationships with AD. Moreover, the model's performance, which was trained and tested using different datasets, confirmed the potential value of hub genes as biomarkers of AD. Taken together, our findings suggest that the identified hub genes are likely to be key players in the pathogenesis of AD and may have potential as therapeutic targets or diagnostic biomarkers.

### 3.5. Ensemble machine learning

**Figure 5:**
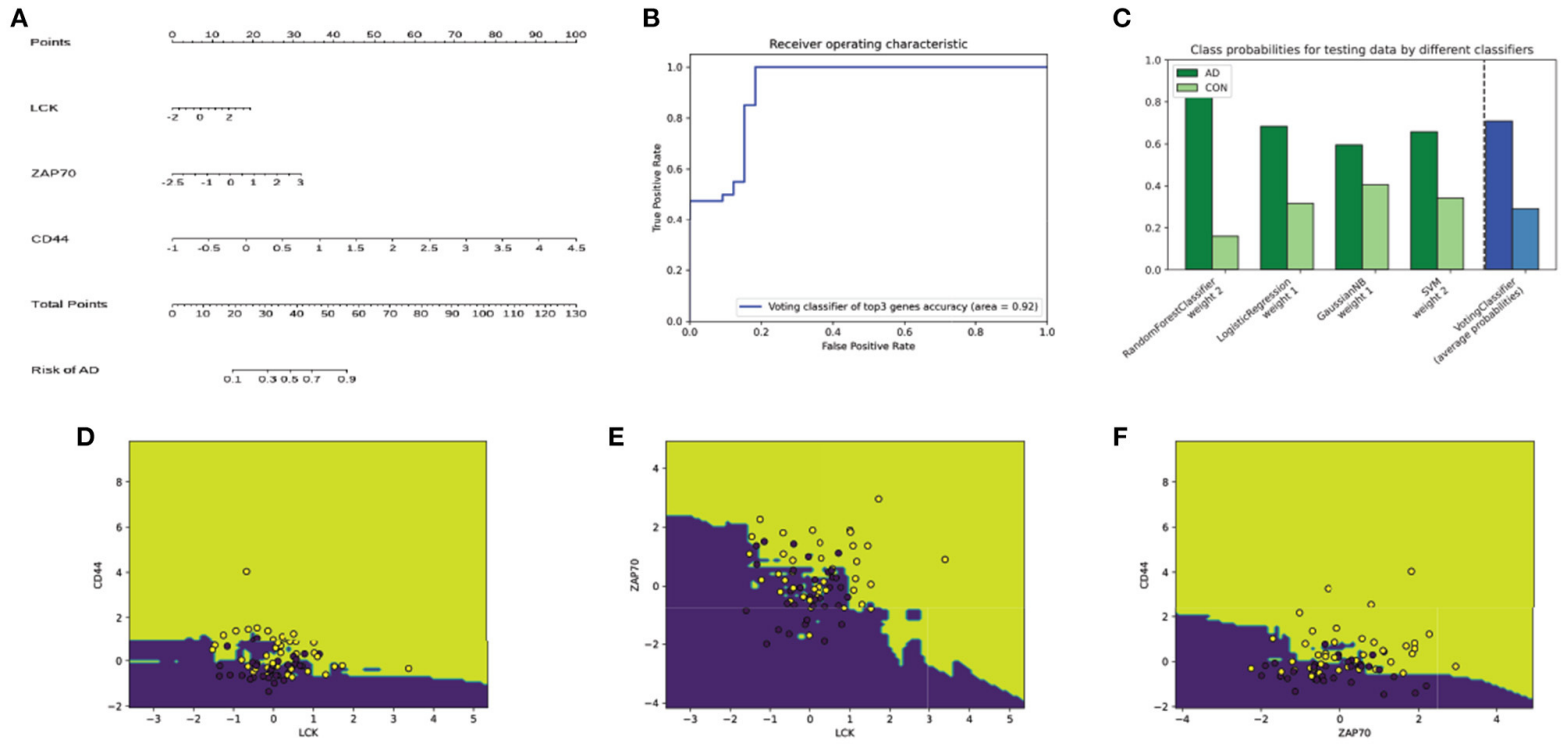
Visualization of ensemble classifier model. **(A)** The nomogram can be used to predict the risk of AD according to the expression of top hub genes. **(B)** The ROC of the model; the area under the ROC is 0.92 representing the accuracy of the model. **(C)** The green bar represents predicted probabilities for each model, and the blue bar represents the ensemble model. Its distribution is generally consistent in every single model. **(D–F)** The decision boundaries of the ensemble model with *CD44* and *LCK* **(D)**, *ZAP70* and *LCK* **(E)**, *CD44* and *ZAP70* **(F)** as input. Dark yellow dots are the samples predicted as AD, whereas dark blue dots are the samples predicted as non-AD. The dark yellow and dark blues areas represent AD and non-AD predicted space, respectively.

We trained an ensemble machine learning classifier consisting of RF, GMM, LM, and SVM binary models (RF) to further identify the top hub genes, *LCK, ZAP70*, and *CD44*, using a higher diagnostic value based on the 80% data of GSE15222. A nomogram that estimates AD risk according to the results predicted by the ensemble model is shown in Figure 5A. The AUC, which represents the accuracy of the model, was 0.92, confirming that it may be reliably used to distinguish between AD and non-AD groups (Figure 5B). The predicted probabilities for each model (green bar), and the classifier probabilities for the ensemble model (blue bar) are shown (Figure 5C). This indicated that the results of each model were coincident. Subsequently, we plotted the decision boundaries for every two genes, wherein a dot represents the predicted result; the surface is the decision space of the model; and the yellow and purple represent AD and non-AD, respectively (Figures 5D–F).

### 3.6. Eigenvalue decomposition analysis

**Figure 6:**
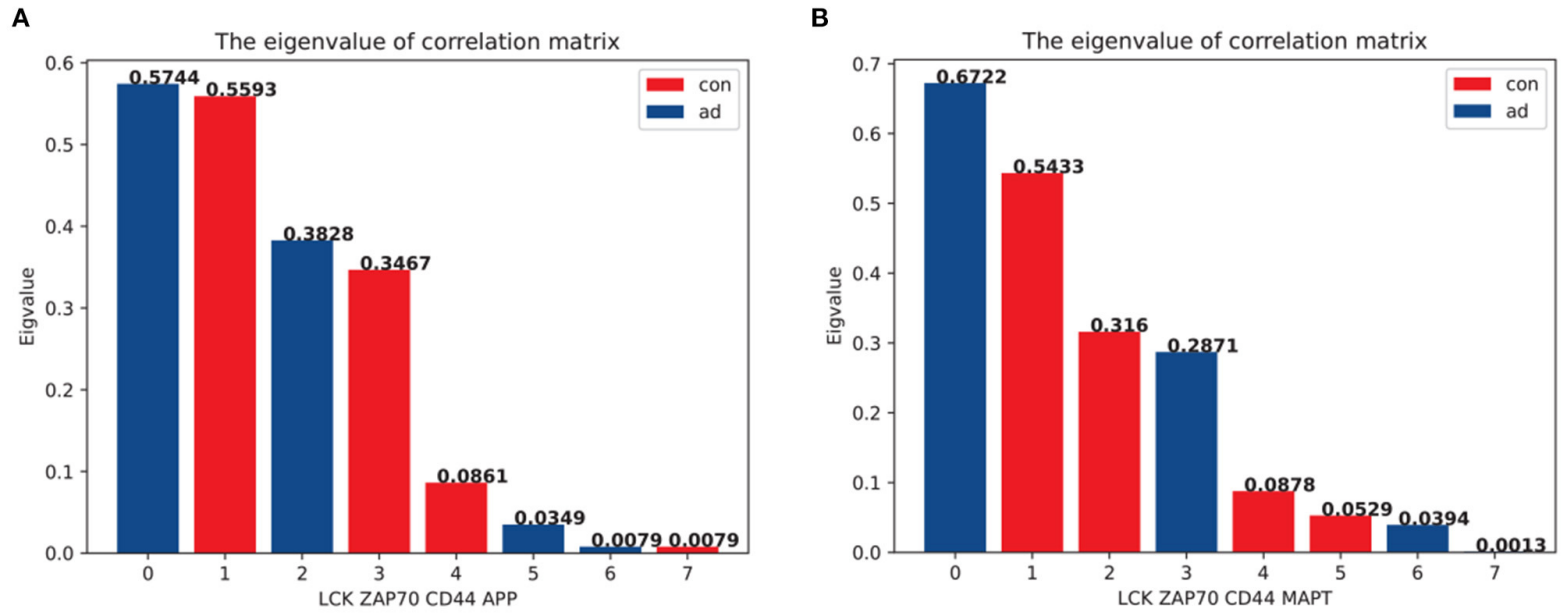
The eigenvalue of the inner product matrix of the top three hub genes. The eigenvalue has been standardized for the purpose of comparison in a different matrix. The blue bar represents the eigenvalue in the AD expression matrix, whereas the red bar represents the eigenvalue in the control group expression matrix. **(A)** The eigenvalues based on the matrix were representative of *LCK*,*ZAP70*,*CD44* and *APP*. **(B)** The eigenvalues based on the matrix were representative of *LCK*,*ZAP70*,*CD44*, and *MAPT*.

In this section, we proposed a novel eigenvalue decomposition method and applied it to the GSE97760 microarray dataset to further investigate the coordination between the top hub genes and AD pathologies. We first calculated the matrix of the inner products of hub genes with *APP* and *MAPT* to determine the changes in eigenvalues between the AD and normal groups. We observed a slight change in the eigenvalues consisting of hub genes and *APP* between the AD and normal groups (Figure 6A). However, when hub genes were combined with *MAPT*, the eigenvalues changed significantly (Figure 6B). The largest eigenvalue changed from 0.543 (in the non-AD group) to 0.672 (in the AD group), while the smallest eigenvalue changed from 0.0013 (non- AD group) to 0.0529 (AD group).

A comparison between Figures 6A, B indicated that hub gene expression was incongruent with the tauopathies associated with AD pathology. This suggests that the interaction between *LCK, ZAP70*, and *CD44* may be involved in NFT formation, which is a characteristic feature of AD pathology. Our analysis of the GSE97760 dataset using our novel eigenvalue decomposition method suggests that the top three hub genes may be involved in tauopathies associated with AD, rather than Aβ pathology. However, further validation and investigation are needed to confirm the involvement of these hub genes in the pathogenesis of AD and to elucidate the precise mechanisms underlying their roles. Nevertheless, our findings provide new insights into the coordination between hub genes and AD pathology, which may aid in the development of new therapeutic strategies for AD.

### 3.7. Protein-protein docking simulation

In this section, we aimed to investigate the potential role of hub genes in Alzheimer's disease (AD) drug design. To this end, we downloaded the Protein Data Bank (PDB) structure files of LCK (ID: P06239), CD44 (ID: P16070), and ZAP70 (ID: P43403), and two choline-related genes, ACHE (ID: P22303) and BCHE (ID: P06276) from the Alphafold2 database. We then used the docking algorithm with each hub gene as a receptor and each choline gene as a ligand, which was applied using Discover Studio 2019.

**Figure 7:**
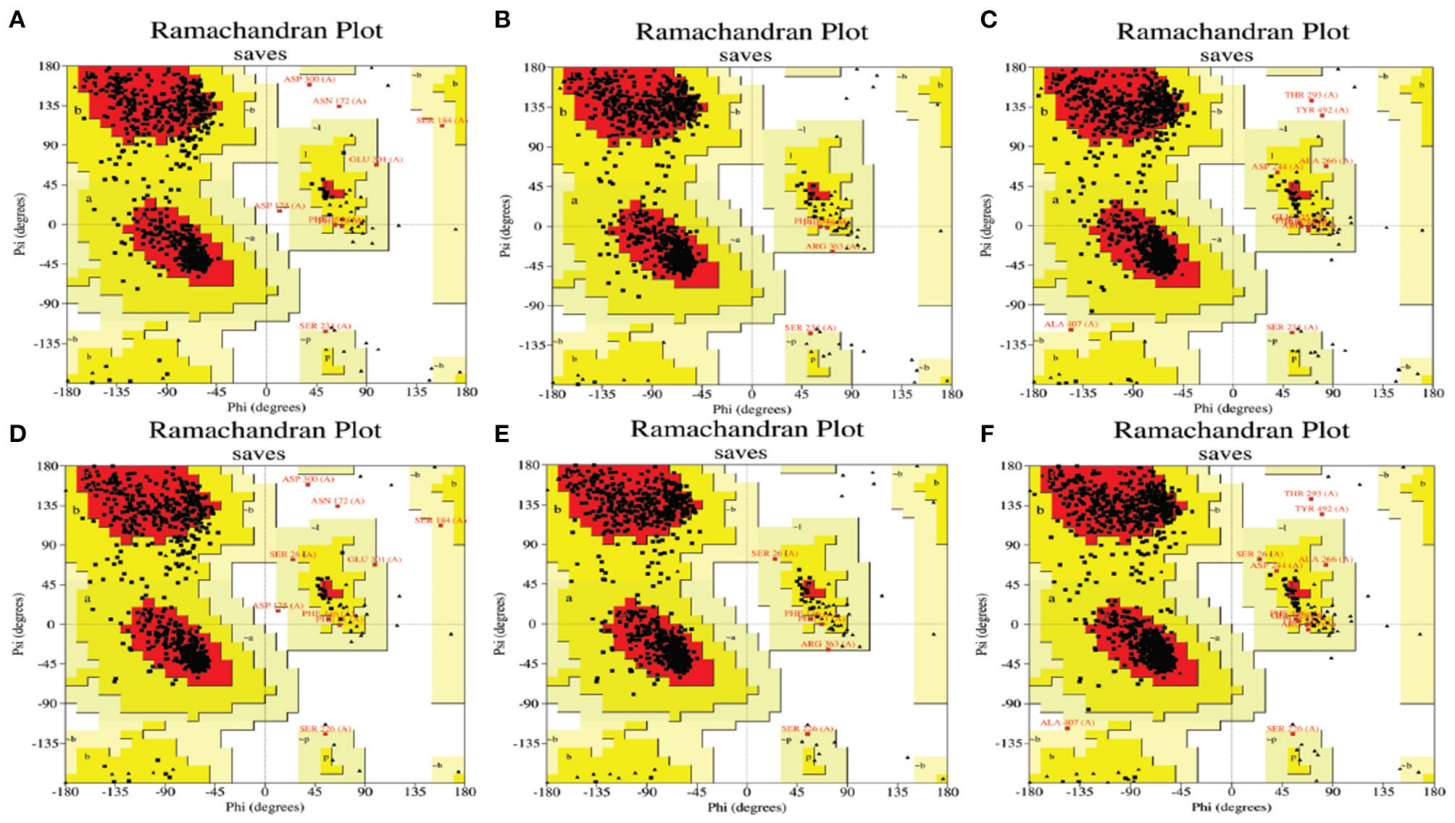
The Ramachandran plot for docking models. The higher number of dots in the red area represent the increased reliability of the model. The models for CD44 and ACHE **(A)**, LCK and ACHE **(B)**, ZAP70 and ACHE **(C)**, CD44 and BCHE **(D)**, LCK and BCHE **(E)**, and ZAP70 and BCHE **(F)**.

After performing the docking analysis, we calculated the docking and confidence scores, and Figure 7 was used to compute the residues in the most favored regions (Table 3). The docking scores of hub genes and ACHE were found to be slightly lower than those of BCHE. We observed that the best predictive model was CD44 and ACHE, which had a –312.09 docking score, a confidence score of 0.9624, and 86.0% of residues in the most favored regions. Our comprehensive analysis revealed that CD44 may play a potential role in AD drug design since it was able to form stable binding sites with ACHE. Thus, CD44 could be a potential drug target for the treatment of AD.

**Table 3:** The result of protein docking between hub genes and choline.

**Receptor**	**Ligand**	**Docking score^a^**	**Confidence score^b^**	**Residues in most favored regions**
LCK	ACHE	–280.67	0.9178	91.90%
CD44	ACHE	–322.09	0.9624	86.00%
ZAP70	ACHE	–293.82	0.9225	89.80%
LCK	BCHE	–275.38	0.9247	91.10%
CD44	BCHE	–265.2	0.9092	85.50%
ZAP70	BCHE	–254.83	0.8906	89.30%

a Docking Score: Docking score represent the possibility of binding. A more negative docking score means a more possible binding model.

b Confidence Score: Represent the binding likeliness of two proteins, the receptor and ligand would be very likely to bind if the confidence score is above 0.7.

## 4. Discussion

AD is the most common disease among the elderly, and its worldwide prevalence is increasing substantially (42). According to the annual report of the AD association, the number of patients with AD is expected to increase from 65.7 million in 2030 to 115.4 million in 2050 (43). Currently, drugs that target AD do not exhibit adequate efficacy owing to the complicated pathology of AD (44). Thus, there is an urgent need to identify potential biomarkers of AD that may help reduce the disease burden. The development and increased use of NGS have helped enhance gene expression profiling (45). One of the most critical applications of NGS is the identification of DEGs because these can be used as “biomarkers,” which reveal the status of a drug response, or “drug targets” if directly associated with drugs (46).

In this study, we used the “limma” package of the R language to analyse the GSE173955 and GSE203206 datasets and identified 623 DEGs between AD patients and normal groups. Next, GO and KEGG enrichment analyses were applied to investigate the biological functions and pathological pathways *via* "clusterProfiler" in the R package. The signal release process and neuroactive ligand-receptor interaction pathway were enriched in the highest number of DEGs (Figure 2). Next, 10 hub genes, namely *LCK, ZAP70, CD44, CD2, SNAP25, CD3E, CXCL8, HIST1H3j, IL12RB2*, and *STAT4*, were identified as DEGs in the PPI network, which was constructed using the STRING database. Among these, *LCK, ZAP70*, and *CD44* were associated with APOE or APP proteins and strongly correlated with common AD pathology. A previous study has confirmed that APP and MAPT may play substantial roles in neuroinflammation (47–50). We used the proposed novel eigenvalue decomposition method to analyse another dataset, GSE97760, to identify the correlation between hub genes and APP or MAPT. The eigenvalue changed significantly when hub genes were integrated with MAPT, compared to APP (Figure 6). This suggested that neuroinflammation pathology involving *LCK, ZAP70*, and *CD44* would affect *MAPT* more than *APP*. Further investigation is required to elucidate the mechanistic interactions responsible for the effect of *MAPT* and hub genes in AD pathogenesis.

Moreover, GSE15222 is an Alzheimer's NGS dataset with 187 non-AD samples and 176 AD cases, of which 80% was used to train models and 20% was used to test the classifier. The single random forest test result (Figure 4) shows that the prediction accuracy (0.84) obtained using all genes as the input of the classifier was only slightly higher compared to that (0.78) obtained using *LCK, ZAP70*, and *CD44* as input. An ensemble model was established to further explore the predictive value of the hub genes. A nomogram was plotted according to the predicted label of the ensemble model (Figure 5), which is easy to use in clinical practice (51). These results implied that the neuroinflammation-related genes, *LCK, ZAP70*, and *CD44*, were strongly associated with AD and were not modified by the changes in the experiments. Thus, they show potential as promising biomarkers of AD.

Lymphocyte-specific protein tyrosine kinase (LCK), encoded by *lck*, is a protein tyrosine kinase that binds to CD4 and CD8 molecules and plays a vital role in T cell development and activation (52). Regulation of LCK activity depends on conformational changes at the plasma membrane induced by phosphorylation. Intracellular signaling pathways activated by the phosphorylation of LCK are essential for T cell differentiation and the release of allergenic cytokines that activate nuclear factor kappa-B (NF-κB) and cytokine IL4/5/13, which cause neuroinflammation and oxidative stress, respectively (53). Previous studies have shown that neurodegenerative diseases, including AD, are mediated by inflammation and neurotoxic factors, such as interleukin-1beta (IL-1β), tumor necrosis factor-alpha (TNF-α), reactive oxygen species (ROS), and NFκB activation (54). In addition, LCK plays a crucial role in initiating TCR proximal signaling events (55).

Cluster of differentiation-44 (CD44) is a cell surface transmembrane glycoprotein with various biological functions. It has been widely implicated as a marker of cancer stem cells (CSC) in several cancers (56). CD44, which partly provides costimulatory signaling in the activation of T cells, may also stimulate the proliferation of T cells (57, 58). Additionally, it is an inflammation-related protein involved in inflammation-induced neurodegenerative ailments, such as AD. Furthermore, recent studies have revealed that CD44, which strongly interacts with TCR, is involved in T cell activation (59–61). Studies have also indicated that CD44 may contribute to the development of AD, and that inhibition of CD44 may serve as a novel neuroprotective treatment strategy against this disease.

Tyrosine protein kinase (ZAP70) is expressed in T cells and stimulates T cell activation and function by interacting with the TCR/CD3 complex. LCK promotes ZAP70 phosphorylation through TCR activation when T cells are stimulated (62). Studies have demonstrated that ZAP70 is an essential protein kinase involved in several signaling pathways that regulate T lymphocyte development and function. Down-regulation of ZAP70 leads to immunodeficiency, with particular reference to T-lymphocyte-mediated immunity, which is often dysfunctional in AD. Aberrant T cells secrete proinflammatory factors and glial cells that cause neuroinflammation in the brain (63). Pathological hallmarks of AD have been linked to the immune system, indicating that T cells play a substantial role in AD (64).

Taken together, interactions between *LCK, ZAP70*, and *CD44* play a role in neuroinflammation and the TCR signaling cascade. Previous studies have shown that neuroinflammation strongly affects ACHE and BCHE (65–67). The signaling cascade of T cells is involved in the late step of ACHE activation (68). TCR activates the mitogen-activated protein kinase (MAPK) pathways, resulting in enhanced ACHE activity. These findings are consistent with our protein-protein docking simulation results (69, 70). The average score and confidence score of ACHE as a ligand were significantly better than those of BCHE (Table 3 and Figure 7). Therefore, we postulate that neuroinflammation and TCR- related proteins may form a more stable docking model with ACHE than with BCHE. These interactions could be considered therapeutic targets in AD.

Despite the encouraging results obtained in this study, several limitations need to be considered. One of the primary limitations is the relatively small sample size in some of the datasets, which could lead to biased estimates and limit the generalizability of the findings. Furthermore, AD is a complex and heterogeneous disease with diverse clinical and pathological phenotypes, and it is possible that the identified DEGs may not be representative of the entire AD population. Moreover, molecular differences in brain regions and cell types could influence gene expression patterns, which might complicate the interpretation of results. It should be emphasized that there is an uneven distribution of age between AD and control participants in certain datasets, which may pose a potential confounding factor. Although we have made efforts to address this concern in the DEG model, it is important to acknowledge the possibility of age bias affecting the results.

Overall, the findings of this study suggest that neuroinflammation and TCR signaling may have a significant impact on the development of Alzheimer's disease. The analysis of hub genes indicated that *LCK, ZAP70*, and *CD44* proteins may play an essential role in this process. The proposed eigenvalue analysis revealed that there is a lack of stability in the coordination between hub genes and *MAPT*, which is a gene that codes for the tau protein involved in AD pathogenesis. This instability implies that the hub genes and MAPT might have a considerable impact on the activity of ACHE, a critical protein involved in AD drug design.

## 5. Conclusions

In summary, this study used integrated bioinformatics tools and datasets to reveal that significant hub genes, such as *LCK, ZAP70, CD44, CD2, SNAP25, CD3E, CXCL8, HIST1H3J, IL12RB2*, and *STAT4*, may play a pivotal role in AD development. The Cytohubba plugin identified *LCK, ZAP70*, and *CD44* as the top three hub genes among all hub genes, as well as neuroinflammation and TCR-related genes. Next, they were verified using the AD database and machine-learning models with credible results. Eigenvalue analysis revealed an imbalance between the top three hub genes and *MAPT* expression. Protein docking showed that LCK, ZAP70, and CD44 could form reliable binding sites with ACHE. Therefore, the top three hub genes may play vital roles in designing drugs based on ACHE inhibitors. However, further studies may be required to fully predict the underlying molecular mechanisms.

## Data availability statement

The original contributions presented in the study are included in the article/Supplementary material, further inquiries can be directed to the corresponding authors.

## Author contributions

Conceptualization: WG, XG, CP, YW, and XZ. Formal analysis, investigation, and writing—original draft: WG. Funding acquisition: YW. Methodology: WG, XG, and QZ. Project administration: CP, YW, and XZ. Supervision: XG and CP. Docking simulation: MP and WG. Validation: LY and QZ. Visualization: WG and LY. Writing—review and editing: XG, PY, and XP. All authors contributed to the article and approved the submitted version.

## References

[1] Jellinger KA Janetzky B Attems J Kienzl E. Biomarkers for early diagnosis of Alzheimer disease:‘ALZheimer ASsociated gene’-a new blood biomarker? J Cell Mol Med. (2008) 12:1094–117. 10.1111/j.1582-4934.2008.00313.x 18363842 PMC3865653

[2] World Health Organization. Diet, Nutrition, and the Prevention of Chronic Diseases: Report of a Joint WHO/FAO Expert Consultation, Vol. 916. World Health Organization (2003). 12768890

[3] Hirtz D Thurman DJ Gwinn-Hardy K Mohamed M Chaudhuri A Zalutsky R. How common are the “common” neurologic disorders? Neurology. (2007) 68:326–37. 10.1212/01.wnl.0000252807.38124.a3 17261678

[4] Pang C Yang H Hu B Wang S Chen M Cohen DS. Identification and analysis of Alzheimer's candidate genes by an amplitude deviation algorithm. J Alzheimers Dis Parkinsonism. (2019) 9:460. 10.4172/2161-0460.1000460 31080696 PMC6505709

[5] Laurent C. Buée L, Blum D. Tau and neuroinflammation: what impact for Alzheimer's Disease and Tauopathies? Biomed J. (2018) 41:21–33. 10.1016/j.bj.2018.01.003 29673549 PMC6138617

[6] Zhang F Jiang L. Neuroinflammation in Alzheimer's disease. Neuropsychiatr Dis Treat. (2015) 11:243. 10.2147/NDT.S75546 25673992 PMC4321665

[7] Wang S Jiang W Ouyang T Shen XY Wang F Qu Yh. Jatrorrhizine balances the gut microbiota and reverses learning and memory deficits in APP/PS1 transgenic mice. Sci Rep. (2019) 9:1–15. 10.1038/s41598-019-56149-9 31862965 PMC6925119

[8] Toublet FX Lecoutey C Lalut J Hatat B Davis A Since M. Inhibiting acetylcholinesterase to activate pleiotropic prodrugs with therapeutic interest in Alzheimer's disease. Molecules. (2019) 24:2786. 10.3390/molecules24152786 31370232 PMC6696315

[9] Sonde L Johnell K. Is drug treatment for dementia followed up in primary care? A Swedish study of dementia clinics and referring primary care centres. PLoS ONE. (2013) 8:e57161. 10.1371/journal.pone.0057161 23437334 PMC3577769

[10] Bott N Kumar S Krebs C Glenn JM Madero EN Juusola JL. A remote intervention to prevent or delay cognitive impairment in older adults: design, recruitment, and baseline characteristics of the Virtual Cognitive Health (VC Health) study. JMIR Res Protoc. (2018) 7:e11368. 10.2196/11368 30104186 PMC6111147

[11] van den Dungen P Moll van Charante EP van de Ven PM van Marwijk HW van der Horst HE van Hout HP. Case finding of mild cognitive impairment and dementia and subsequent care; results of a cluster RCT in primary care. PLoS ONE. (2016) 11:e0156958. 10.1371/journal.pone.0156958 27310616 PMC4910994

[12] Giau VV Bagyinszky E Yang YS Youn YC An SSA Kim SY. Genetic analyses of early-onset Alzheimer's disease using next generation sequencing. Sci Rep. (2019) 9:1–10. 10.1038/s41598-019-44848-2 31182772 PMC6557896

[13] Singh HN Swarup V Dubey NK Jha NK Singh AK Lo WC. Differential transcriptome profiling unveils novel deregulated gene signatures involved in pathogenesis of Alzheimer's disease. Biomedicines. (2022) 10:611. 10.3390/biomedicines10030611 35327413 PMC8945049

[14] Ling J Yang S Huang Y Wei D Cheng W. Identifying key genes, pathways and screening therapeutic agents for manganese-induced Alzheimer disease using bioinformatics analysis. Medicine. (2018) 97:e10775. 10.1097/MD.0000000000010775 29851783 PMC6392515

[15] Mizuno Y Abolhassani N Mazzei G Sakumi K Saito T Saido TC. MUTYH actively contributes to microglial activation and impaired neurogenesis in the pathogenesis of Alzheimer's disease. Oxid Med Cell Longev. (2021) 2021:8635088. 10.1155/2021/8635088 34970419 PMC8714343

[16] Caldwell AB Anantharaman BG Ramachandran S Nguyen P Liu Q Trinh I. Transcriptomic profiling of sporadic Alzheimer's disease patients. Mol Brain. (2022) 15:1–7. 10.1186/s13041-022-00963-2 36224601 PMC9559068

[17] Robasky K Lewis NE Church GM. The role of replicates for error mitigation in next-generation sequencing. Nat Rev Genet. (2014) 15:56–62. 10.1038/nrg3655 24322726 PMC4103745

[18] Webster JA Gibbs JR Clarke J Ray M Zhang W Holmans P. Genetic control of human brain transcript expression in Alzheimer disease. Am J Hum Genet. (2009) 84:445–58. 10.1016/j.ajhg.2009.03.011 19361613 PMC2667989

[19] Chen S Zhou Y Chen Y Gu J. fastp: an ultra-fast all-in-one FASTQ preprocessor. Bioinformatics. (2018) 34:i884–90. 10.1093/bioinformatics/bty560 30423086 PMC6129281

[20] Law CW Chen Y Shi W Smyth GK. voom: Precision weights unlock linear model analysis tools for RNA-seq read counts. Genome Biol. (2014) 15:1–17. 10.1186/gb-2014-15-2-r29 24485249 PMC4053721

[21] Ritchie ME Phipson B Wu D Hu Y Law CW Shi W. limma powers differential expression analyses for RNA-sequencing and microarray studies. Nucleic Acids Res. (2015) 43:e47. 10.1093/nar/gkv007 25605792 PMC4402510

[22] Phipson B Lee S Majewski IJ Alexander WS Smyth GK. Robust hyperparameter estimation protects against hypervariable genes and improves power to detect differential expression. Ann Appl Stat. (2016) 10:946. 10.1214/16-AOAS920 28367255 PMC5373812

[23] Young MD Wakefield MJ Smyth GK Oshlack A. Gene ontology analysis for RNA-seq: accounting for selection bias. Genome Biol. (2010) 11:1–12. 10.1186/gb-2010-11-2-r14 20132535 PMC2872874

[24] Kanehisa M Goto S Furumichi M Tanabe M Hirakawa M. KEGG for representation and analysis of molecular networks involving diseases and drugs. Nucleic Acids Res. (2010) 38(Suppl_1):D355–60. 10.1093/nar/gkp896 19880382 PMC2808910

[25] Chin CH Chen SH Wu HH Ho CW Ko MT Lin CY. cytoHubba: identifying hub objects and sub-networks from complex interactome. BMC Syst Biol. (2014) 8:S11. 10.1186/1752-0509-8-S4-S11 25521941 PMC4290687

[26] Xu M Zhang DF Luo R Wu Y Zhou H Kong LL. A systematic integrated analysis of brain expression profiles reveals YAP1 and other prioritized hub genes as important upstream regulators in Alzheimer's disease. Alzheimers Dement. (2018) 14:215–29. 10.1016/j.jalz.2017.08.012 28923553

[27] Breiman L. Random forests. Mach Learn. (2001) 45:5–32. 10.1023/A:1010933404324

[28] Sagi O Rokach L. Ensemble learning: a survey. Wiley Interdiscipl Rev Data Min Knowl Discov. (2018) 8:e1249. 10.1002/widm.1249

[29] Pedregosa F Varoquaux G Gramfort A Michel V Thirion B Grisel O. Scikit-learn: machine learning in Python. J Mach Learn Res. (2011) 12:2825–30.

[30] Orr MJ. Introduction to Radial Basis Function Networks. Technical Report. Center for Cognitive Science; University of Edinburgh (1996).

[31] Kumari S Kumar D Mittal M. An ensemble approach for classification and prediction of diabetes mellitus using soft voting classifier. Int J Cogn Comput Eng. (2021) 2:40–6. 10.1016/j.ijcce.2021.01.001

[32] Sherazi SWA Bae JW Lee JY. A soft voting ensemble classifier for early prediction and diagnosis of occurrences of major adverse cardiovascular events for STEMI and NSTEMI during 2-year follow-up in patients with acute coronary syndrome. PLoS ONE. (2021) 16:e0249338. 10.1371/journal.pone.0249338 34115750 PMC8195401

[33] Akram P Liao L. Prediction of comorbid diseases using weighted geometric embedding of human interactome. BMC Med Genomics. (2019) 12:161. 10.1186/s12920-019-0605-5 31888634 PMC6936100

[34] Iasonos A Schrag D Raj GV Panageas KS. How to build and interpret a nomogram for cancer prognosis. J Clin Oncol. (2008) 26:1364–70. 10.1200/JCO.2007.12.9791 18323559

[35] Secades JJ Frontera G. CDP-choline: pharmacological and clinical review. Methods Find Exp Clin Pharmacol. (1995) 17:1–54. 8709678

[36] Parnetti L Amenta F Gallai V. Choline alphoscerate in cognitive decline and in acute cerebrovascular disease: an analysis of published clinical data. Mech Ageing Dev. (2001) 122:2041–55. 10.1016/S0047-6374(01)00312-8 11589921

[37] Moreno MDJM. Cognitive improvement in mild to moderate Alzheimer's dementia after treatment with the acetylcholine precursor choline alfoscerate: a multicenter, double-blind, randomized, placebo-controlled trial. Clin Ther. (2003) 25:178–93. 10.1016/S0149-2918(03)90023-3 12637119

[38] Dominguez C Boelens R Bonvin AM. HADDOCK: a protein to protein docking approach based on biochemical or biophysical information. J Am Chem Soc. (2003) 125:1731–7. 10.1021/ja026939x 12580598

[39] Jumper J Evans R Pritzel A Green T Figurnov M Ronneberger O. Highly accurate protein structure prediction with AlphaFold. Nature. (2021) 596:583–9. 10.1038/s41586-021-03819-2 34265844 PMC8371605

[40] Varadi M Anyango S Deshpande M Nair S Natassia C Yordanova G. AlphaFold Protein Structure Database: massively expanding the structural coverage of protein-sequence space with high-accuracy models. Nucleic Acids Res. (2022) 50:D439–44. 10.1093/nar/gkab1061 34791371 PMC8728224

[41] Kleywegt GJ Jones TA. Phi/psi-chology: Ramachandran revisited. Structure. (1996) 4:1395–400. 10.1016/S0969-2126(96)00147-5 8994966

[42] Agarwal S Ghanty P Pal NR. Identification of a small set of plasma signalling proteins using neural network for prediction of Alzheimer's disease. Bioinformatics. (2015) 31:2505–13. 10.1093/bioinformatics/btv173 25819077

[43] Zeisel J Bennett K Fleming R. World Alzheimer Report 2020: Design, Dignity, Dementia: Dementia-Related Design and the Built Environment. (2020).

[44] Zhang W Zhang Y Hu N Wang A. Alzheimer's disease-associated inflammatory pathways might contribute to osteoporosis through the interaction between PROK2 and CSF3. Front Neurol. (2022) 13:990779. 10.3389/fneur.2022.990779 36203970 PMC9531648

[45] Rodriguez-Esteban R Jiang X. Differential gene expression in disease: a comparison between high-throughput studies and the literature. BMC Med Genomics. (2017) 10:59. 10.1186/s12920-017-0293-y 29020950 PMC5637346

[46] Es HA Mahdizadeh H Asl AHA Totonchi M. Genomic alterations and possible druggable mutations in carcinoma of unknown primary (CUP). Sci Rep. (2021) 11:15112. 10.1038/s41598-021-94678-4 34302033 PMC8302572

[47] Ghosal K Stathopoulos A Pimplikar SW. APP intracellular domain impairs adult neurogenesis in transgenic mice by inducing neuroinflammation. PLoS ONE. (2010) 5:e11866. 10.1371/journal.pone.0011866 20689579 PMC2912762

[48] Heneka MT Carson MJ El Khoury J Landreth GE Brosseron F Feinstein DL. Neuroinflammation in Alzheimer's disease. Lancet Neurol. (2015) 14:388–405. 10.1016/S1474-4422(15)70016-5 25792098 PMC5909703

[49] Bevan-Jones WR Cope TE Jones PS Passamonti L Hong YT Fryer T. In vivo evidence for pre-symptomatic neuroinflammation in a MAPT mutation carrier. Ann Clin Transl Neurol. (2019) 6:373–8. 10.1002/acn3.683 30847369 PMC6389753

[50] Metcalfe MJ Figueiredo-Pereira ME. Relationship between tau pathology and neuroinflammation in Alzheimer's disease. Mount Sinai J Med. (2010) 77:50–8. 10.1002/msj.20163 20101714 PMC2904237

[51] Gondo OT Gondo T Hamada R. Nomogram as predictive model in clinical practice. Gan Kagaku Ryoho. (2009) 36:901–6. 19542708

[52] Bozso SJ Kang JJ Nagendran J. The role of competing mechanisms on Lck regulation. Immunol Res. (2020) 68:289–95. 10.1007/s12026-020-09148-2 32794043

[53] Kim EJ Monje FJ Li L Höger H Pollak DD Lubec G. Alzheimer's disease risk factor lymphocyte-specific protein tyrosine kinase regulates long-term synaptic strengthening, spatial learning and memory. Cell Mol Life Sci. (2013) 70:743–59. 10.1007/s00018-012-1168-1 23007847 PMC11113176

[54] Kempuraj D Thangavel R Natteru P Selvakumar G Saeed D Zahoor H. Neuroinflammation induces neurodegeneration. J Neurol Neurosurg Spine. (2016) 1:1003. 10.33140/JNS 28127589 PMC5260818

[55] Schoenborn JR Tan YX Zhang C Shokat KM Weiss A. Feedback circuits monitor and adjust basal Lck-dependent events in T cell receptor signaling. Sci Signal. (2011) 4:ra59. 10.1126/scisignal.2001893 21917715 PMC4080844

[56] Hassn Mesrati M Syafruddin SE Mohtar MA Syahir A. CD44: a multifunctional mediator of cancer progression. Biomolecules. (2021) 11:1850. 10.3390/biom11121850 34944493 PMC8699317

[57] Galluzzo E Albi N Fiorucci S Merigiola C Ruggeri L Tosti A. Involvement of CD44 variant isoforms in hyaluronate adhesion by human activated T cells. Eur J Immunol. (1995) 25:2932–9. 10.1002/eji.1830251033 7589094

[58] Fiorucci S Mencarelli A Palazzetti B Distrutti E Vergnolle N Hollenberg MD. Proteinase-activated receptor 2 is an anti-inflammatory signal for colonic lamina propria lymphocytes in a mouse model of colitis. Proc Nat Acad Sci USA. (2001) 98:13936–41. 10.1073/pnas.241377298 11717450 PMC61145

[59] Ponta H Sherman L Herrlich PA. CD44: from adhesion molecules to signalling regulators. Nat Rev Mol Cell Biol. (2003) 4:33–45. 10.1038/nrm1004 12511867

[60] Huet S Groux H Caillou B Valentin H Prieur A Bernard A. CD44 contributes to T cell activation. J Immunol. (1989) 143:798–801. 10.4049/jimmunol.143.3.798 2568380

[61] Hegde VL Singh NP Nagarkatti PS Nagarkatti M. CD44 mobilization in allogeneic dendritic cell-T cell immunological synapse plays a key role in T cell activation. J Leukoc Biol. (2008) 84:134–42. 10.1189/jlb.1107752 18388297 PMC3178506

[62] Chen M Chen X Hu Y Cai X. Screening of key genes related to the prognosis of mouse sepsis. Biosci Rep. (2020) 40:BSR20202649. 10.1042/BSR20202649 33015708 PMC7601352

[63] Lee SH Rezzonico MG Friedman BA Huntley MH Meilandt WJ Pandey S. TREM2-independent oligodendrocyte, astrocyte, and T cell responses to tau and amyloid pathology in mouse models of Alzheimer disease. Cell Rep. (2021) 37:110158. 10.1016/j.celrep.2021.110158 34965428

[64] Dai L Shen Y. Insights into T-cell dysfunction in Alzheimer's disease. Aging Cell. (2021) 20:e13511. 10.1111/acel.13511 34725916 PMC8672785

[65] Borchert T Hess A. Lukacević M, Ross TL, Bengel FM, Thackeray JT. Angiotensin-converting enzyme inhibitor treatment early after myocardial infarction attenuates acute cardiac and neuroinflammation without effect on chronic neuroinflammation. Eur J Nucl Med Mol Imaging. (2020) 47:1757–68. 10.1007/s00259-020-04736-8 32125488 PMC7248052

[66] Liu J Li H Gong T Chen W Mao S Kong Y. Anti-neuroinflammatory effect of short-chain fatty acid acetate against Alzheimer's disease via upregulating GPR41 and inhibiting ERK/JNK/NF-κB. J Agric Food Chem. (2020) 68:7152–61. 10.1021/acs.jafc.0c02807 32583667

[67] Kim S Watt T Ceballos N Sharma S. Adverse childhood experiences and neuroinflammatory biomarkers–The role of sex. Stress Health. (2019) 35:432–40. 10.1002/smi.2871 31099473

[68] Jiang H Zhang XJ. Acetylcholinesterase and apoptosis: a novel perspective for an old enzyme. FEBS J. (2008) 275:612–7. 10.1111/j.1742-4658.2007.06236.x 18205833

[69] Zhang XJ Greenberg DS. Acetylcholinesterase involvement in apoptosis. Front Mol Neurosci. (2012) 5:40. 10.3389/fnmol.2012.00040 22514517 PMC3322359

[70] Fujii T Mashimo M Moriwaki Y Misawa H Ono S Horiguchi K. Physiological functions of the cholinergic system in immune cells. J Pharmacol Sci. (2017) 134:1–21. 10.1016/j.jphs.2017.05.002 28552584

