# Corrigendum: Exploring the interaction between T-cell antigen receptor-related genes and MAPT or ACHE using integrated bioinformatics analysis

**DOI:** 10.3389/fneur.2023.1204487

**Published:** 2023-04-26

**Authors:** Wenbo Guo, Xun Gou, Lei Yu, Qi Zhang, Ping Yang, Minghui Pang, Xinping Pang, Chaoyang Pang, Yanyu Wei, XiaoYu Zhang

**Affiliations:** ^1^College of Computer Science, Sichuan Normal University, Chengdu, China; ^2^College of Life Science, Sichuan Normal University, Chengdu, China; ^3^College of Mathematics and Physics, Chengdu University of Technology, Chengdu, China; ^4^West China School of Basic Medical Sciences and Forensic Medicine, Sichuan University, Chengdu, China; ^5^National Key Laboratory of Science and Technology on Vacuum Electronics, School of Electronic Science and Engineering, University of Electronic Science and Technology of China, Chengdu, China

**Keywords:** Alzheimer's disease, RNA sequencing, neuroinflammation, random forest classifier, ensemble learning, T-cell antigen receptor, eigenvalue decomposition, protein docking

In the published article, an author name was incorrectly written as “Yanyun Wei.” The correct spelling is “Yanyu Wei.”

The authors apologize for this error and state that this does not change the scientific conclusions of the article in any way. The original article has been updated.

