# Supplementary material for: Exploring the interaction between T-cell antigen receptor-related genes and MAPT or ACHE using integrated bioinformatics analysis
Source: Front Neurol. 2023 Mar 28;14:1129470. doi: 10.3389/fneur.2023.1129470 (PMC10086260; doi:10.3389/fneur.2023.1129470)
Supplement: Supplementary file 1 [file Table_1.DOCX]

Supplementary Material

Wenbo Guo^1^, Xun Gou^2^ , Lei Yu^1^, Qi Zhang^1^, Ping Yang^1^,Minghui Pang^3^, Xinping Pang^4^, Chaoyang Pang^1*^, Yanyun Wei^5*^, XiaoYu Zhang^2*^

**Correspondence:**

Chaoyang Pang^1*^: cypang@sicnu.edu.cn

Yanyun Wei^5*^ : yywei@uestc.edu.cn

XiaoYu Zhang^2*^ : [zhangxy2005@126.com](mailto:zhangxy2005@126.com)

# Description of convert fasta files to gene expression

In this article, we analysed the GSE173955 RNA NGS data base FASTA raw data. Converting a FASTA file into gene expression involves several steps and the use of specific tools. FASTA files contain nucleotide sequences of DNA or RNA, and gene expression data describes the activity level of genes in a sample. The following is a step-by-step description of how to convert a FASTA file into gene expression data in this article.

**Step 1 - Download the raw data**: The NGS data sets can be very large, often comprising several gigabytes or even terabytes of data. The size of GSE173955 raw data is about 100 GB, which is challenged to download if do not use a reliable download tools. Therefor, ‘Aspera’ tools was used to download the raw data in this article. The Aspera's proprietary transfer protocol can transfer data at very high speeds, up to 100 times faster than traditional transfer methods such as FTP or HTTP. Aspera's transfer protocol includes error detection and correction mechanisms that ensure data is transferred accurately and without corruption. This is especially important when transferring large NGS data sets. The code of using ‘Aspera’ to download first replicates is:

| “ascp -i ~/.aspera/connect/etc/asperaweb_id_dsa.openssh -l 100M -T -P33001 -k 1 era-fasp@fasp.sra.ebi.ac.uk:/vol1/fastq/SRR144/010/SRR14436610/SRR14436610_1.fastq.gz ./  ascp -i ~/.aspera/connect/etc/asperaweb_id_dsa.openssh -l 100M -T -P33001 -k 1 era-fasp@fasp.sra.ebi.ac.uk:/vol1/fastq/SRR144/010/SRR14436610/SRR14436610_2.fastq.gz ./” |
| --- |

Where **‘-T’** disable the encrypt transmission as NCBIs or EBI download server doesn’t offer encryption. **‘-k 1’** enable the resumable transfer. This is important because of the large size of most NGS data. The **1** specifies that a sparse checksum will be performed before resuming a transfer which is the best choice for NGS data because a full checksum on large files may be slow.**“SRR14436610_1.fastq.gz”** is the forward sequencing data of first replicate where **“SRR14436610_2.fastq.gz”** is the reverse reads.

**Step 2 - Filtering the raw data:** Filtering raw NGS data is an essential step in NGS data analysis pipeline. This is because raw NGS data often contains a significant amount of noise, errors, and artifacts that can impact downstream analyses, such as assembly, mapping, and variant calling. Filtering the raw data helps remove these unwanted components and improves the quality of the data, making it more reliable and informative for downstream analyses.

‘Fastap’is one of the software tools commonly used to filter NGS data. It is a program designed to filter raw DNA sequence data, including Illumina and PacBio data. In this article, "Fastp" (version 0.232, <https://github.com/OpenGene/fastp>) software was used to trim and filter raw reads. The code for filter the first replicates of GSE173955 into clean data as follows:

| fastp -l 50 -Y 30 -e 20 -i SRR14436589_1.fastq.gz -I SRR14436589_2.fastq.gz -o AD1_1.R1.fastq.gz -O AD1_1.R2.fastq.gz |
| --- |

Where **“-l 50”** is cut the reads if the lengths below 50 bp, **“-Y 30”** and **“-e 20”** is filter the reads if the complexities below 30% and mean quality scores below 20. Moreover, the name of clean data files has changed to **“AD1_1.R1.fastq.gz”** and **“AD1_1.R2.fastq.gz”** for clear understanding in follow steps.

**Step 3 – Mapping**

To map the nucleotide sequences to their corresponding genes, we need a reference genome. The reference genome is a collection of all the genes in an organism, and it provides a map of the location of each gene in the genome. In this article, we used the 'Hisat2' (version 2.2.1) tool to map the GSE173955 dataset to the Hg19 reference genome available on Ensemble (accessible via <https://ftp.ensembl.org/pub/grch37/current/>). The code for mapping as below:

| hisat2 -p 15 –dta -x hg19.fa -1 AD1_1.R1.fastq.gz -2 AD1_1.R2.fastq.gz \| samtools sort -@ 15 -o AD1_1.bam |
| --- |

where **‘-p 15’** represents use 15 cores to run this code. **‘-dta’** parameter led to fewer alignments with short anchors, leading to a significant improvement in computational efficiency and memory usage for transcript assemblers. Moreover, we did not directly save the results of ‘Hisat2’ but use the ‘samtools’ to sort the results and save them in bam file. **‘-@ 15’** enable samtools use 15 cores and ‘**-o AD1_1.bam’** is the sorted bam file of first replicate.

**Step 4 – Quantification**

To quantify gene expression, we need to count the number of reads that map to each gene. In this article we use ‘StringTie’ (version v2.2.0) tool to assemble the GSE173955. The following code uses ‘StringTie’ to count the number of reads that map to each gene:

| stringtie -p 15 -e -B -G hg19.gtf -A gene_abundances.tsv -o transcripts.gtf AD1_1.bam |
| --- |

where **‘-p 15’** represents use 15 cores to run this code. **‘-e’** enable StringTie to run in the expression estimation mode, this is important for samples from hum. It also limits the processing of read alignments to only estimate and output assembled transcripts that match the reference transcript given with the **‘-G’** option. With this option, processing of assembled transcripts that do not match the reference transcript is skipped, which greatly improves processing speed. **‘-A gene_abundances.tsv’** is the gene abundance file. **‘-o transcripts.gtf’** is the merged gtf file. The **'AD1_1.bam'** file serves as the input file for which samtools has performed a sort.

**Step 4 – Normalization**

In order to compare gene expression across samples, it is necessary to normalize the gene expression data. In this article, we utilized the **Voom** approach based on Trimmed Mean of M values **(TMM)** normalization to quantify gene expression. This method corrects for technical variability in RNA-seq data that can arise from differences in sequencing depth, library composition, and other sources of systematic variation. The **TMM** normalization ensures that read counts are comparable between samples, while the **Voom** transformation accounts for the mean-variance relationship of the data, which is crucial for downstream analysis, such as differential expression analysis. Moreover, according to section 15 of the 'Limma' manual on RNA-Seq Data, when there is significant variation in library sizes among samples, the Voom approach is statistically more robust. Supplementary Table 2 shows that the range of clean reads for GSE173955 varies greatly, ranging from 12,506,358 to 46,876,658. Thus, we employed the '**voom**' function based on **TMM** normalization of ‘Limma’ Packages (version 3.48.3) in R (R version 4.1.0 (2021-05-18)). The core R codes as below:

| library(limma)  library(edgeR)  countData <- read.table("counts.txt", header=TRUE, row.names=1)  design_matrix <- read.csv(‘design.csv’)  keep <- filterByExpr(dge, design= design_matrix)  dge <- dge[keep,,keep.lib.sizes=FALSE]  dge <- calcNormFactors(dge)  v <- voom(counts=dge, design=design_matrix) |
| --- |

The **‘counts.txt’** file containing the read count data for GSE173955 was filtered using the **‘filterByExpr’** function to remove rows with consistently zero or very low counts. The variable **‘design_matrix’** is a four-column design matrix that included disease status, age, and gender as covariates. The **‘calcNormFactors’** function was then used to calculate the TMM normalization values for GSE173955. Finally, the **‘voom’** function was applied to calculate the final normalization values.

Please note that the code provided above represents only the core processing steps for GSE173955. The full set of Perl, R, Python code, and Linux commands used in the analysis can be found at <https://github.com/guowenbo1/deg_rfc>.

**Supplementary Table 1.** The design matrix used in Limma.

| **Sample** | **Group** | **Age** | **Gender** |
| --- | --- | --- | --- |
| AD1_1 | AD | 88 | Female |
| AD1_2 | AD | 88 | Female |
| AD2_1 | AD | 95 | Female |
| AD2_2 | AD | 95 | Female |
| AD3_1 | AD | 95 | Female |
| AD3_2 | AD | 95 | Female |
| AD4_1 | AD | 100 | Female |
| AD4_2 | AD | 100 | Female |
| AD5_1 | AD | 99 | Male |
| AD5_2 | AD | 99 | Male |
| AD6_1 | AD | 83 | Male |
| AD6_2 | AD | 83 | Male |
| AD7_1 | AD | 90 | Male |
| AD7_2 | AD | 90 | Male |
| AD8_1 | AD | 84 | Female |
| AD8_2 | AD | 84 | Female |
| Con1_1 | Con | 87 | Female |
| Con1_2 | Con | 87 | Female |
| Con2_1 | Con | 80 | Female |
| Con2_2 | Con | 80 | Female |
| Con3_1 | Con | 84 | Female |
| Con3_2 | Con | 84 | Female |
| Con4_1 | Con | 77 | Male |
| Con4_2 | Con | 77 | Male |
| Con5_1 | Con | 55 | Male |
| Con5_2 | Con | 55 | Male |
| Con5_3 | Con | 55 | Male |
| Con5_4 | Con | 55 | Male |
| Con6_1 | Con | 72 | Female |
| Con6_2 | Con | 72 | Female |
| Con7_1 | Con | 78 | Female |
| Con7_2 | Con | 78 | Female |
| Con8_1 | Con | 83 | Male |
| Con8_2 | Con | 83 | Male |
| Con9_1 | Con | 80 | Male |
| Con9_2 | Con | 80 | Male |
| Con10_1 | Con | 74 | Male |
| Con10_2 | Con | 74 | Male |

Supplementary Table 2. The read information of GSE173955

| Sample | Raw data reads | Clean data reads | Clean data q30 rate |
| --- | --- | --- | --- |
| AD1_1 | 39984102 | 38828726 | 0.954674 |
| AD1_2 | 40606048 | 39435068 | 0.954857 |
| AD2_1 | 12752030 | 12336460 | 0.953883 |
| AD2_2 | 12929478 | 12506358 | 0.954077 |
| AD2_3 | 48361184 | 46625922 | 0.941384 |
| AD2_4 | 48612898 | 46876658 | 0.943818 |
| AD3_1 | 36749632 | 35743780 | 0.95529 |
| AD3_2 | 37307360 | 36290208 | 0.955485 |
| AD4_1 | 31777490 | 30820684 | 0.954157 |
| AD4_2 | 32216348 | 31249750 | 0.954303 |
| AD5_1 | 30715166 | 29461748 | 0.940835 |
| AD5_2 | 30846898 | 29601804 | 0.943244 |
| AD6_1 | 18437670 | 17834806 | 0.945501 |
| AD6_2 | 18696350 | 18090322 | 0.947695 |
| AD7_1 | 38509464 | 37043780 | 0.940206 |
| AD7_2 | 38727860 | 37254866 | 0.94263 |
| AD8_1 | 33145832 | 32222998 | 0.955373 |
| AD8_2 | 33549378 | 32622966 | 0.955526 |
| CON1_1 | 26127178 | 25100210 | 0.941196 |
| CON1_2 | 26261204 | 25243422 | 0.943761 |
| CON2_1 | 32834408 | 31840724 | 0.954635 |
| CON2_2 | 33286174 | 32281118 | 0.954859 |
| CON3_1 | 43650598 | 42419730 | 0.954703 |
| CON3_2 | 44275890 | 43030928 | 0.95489 |
| CON4_1 | 44865386 | 43531934 | 0.954639 |
| CON4_2 | 45422004 | 44073514 | 0.954822 |
| CON5_1 | 19898436 | 19296046 | 0.954288 |
| CON5_2 | 20152382 | 19539744 | 0.954518 |
| CON5_3 | 36215416 | 34904772 | 0.941157 |
| CON5_4 | 36387940 | 35085940 | 0.943683 |
| CON6_1 | 48659342 | 46734662 | 0.940633 |
| CON6_2 | 48908342 | 46977130 | 0.942978 |
| CON7_1 | 34046488 | 32853144 | 0.940016 |
| CON7_2 | 34202644 | 33016956 | 0.942413 |
| CON8_1 | 29957154 | 29045508 | 0.953867 |
| CON8_2 | 30323204 | 29399678 | 0.954048 |
| CON9_1 | 35418852 | 34090802 | 0.938991 |
| CON9_2 | 35485372 | 34166426 | 0.941447 |
| CON10_1 | 31685944 | 30009556 | 0.940508 |
| CON10_2 | 31815996 | 30141544 | 0.942938 |

Supplementary Table 2. The common significant GO pathways of CD44, LCK and ZAP70

| ID | Description | qvalue | geneID |
| --- | --- | --- | --- |
| GO:0007159 | leukocyte cell-cell adhesion | 0.019 | FOXJ1/GLMN/EXT1/THY1/GNRH1/HLA-DQB2/ANXA1/EGR3/IL1RL2/IGF2/ZC3H12A/CD44/CR1/LCK/CCL5/CCR7/BMP4/CD3E/S100A8/CD177/HLA-DOB/ZAP70/IL6/TNF |
| GO:0045785 | positive regulation of cell adhesion | 0.021 | CCDC80/THY1/EPHA4/ECM2/COL8A1/HLA-DQB2/ANXA1/EGR3/SFRP2/IL1RL2/ANGPT1/IGF2/CD44/CR1/LCK/CCL5/NPY2R/CCR7/IBSP/CD3E/LIF/FUT3/HLA-DOB/ZAP70/IL6/TNF |
| GO:0050863 | regulation of T cell activation | 0.032 | FOXJ1/GLMN/THY1/GNRH1/HLA-DQB2/ANXA1/EGR3/IL1RL2/CD2/IGF2/ZC3H12A/CR1/LCK/CCL5/CCR7/CD44/BMP4/CD3E/HLA-DOB/ZAP70/IL6 |

Supplementary Table 3. The matrix of inner product of hub genes with APP in control group.

|  | **LCK** | **ZAP70** | **CD44** | **APP** |
| --- | --- | --- | --- | --- |
| **LCK** | 39.89356353 | 10.67084261 | 0.014896535 | 14.98405594 |
| **ZAP70** | 10.67084261 | 40.72583844 | 0.010609914 | 0.034872322 |
| **CD44** | 0.014896535 | 0.010609914 | 8.25272277 | -0.040865986 |
| **APP** | 14.98405594 | 0.034872322 | -0.04086598 | 6.933966421 |

Supplementary Table 4. The matrix of inner product of hub genes with APP in AD group.

|  | **LCK** | **ZAP70** | **CD44** | **APP** |
| --- | --- | --- | --- | --- |
| **LCK** | 5.727069923 | 0.319953916 | 6.273151048 | -1.257752467 |
| **ZAP70** | 0.319953916 | 0.737007391 | -0.011864764 | -0.124045955 |
| **CD44** | 6.273151048 | -0.011864764 | 7.566364871 | -1.40482008 |
| **APP** | -1.257752467 | -0.124045955 | -1.40482008 | 10.26399852 |

Supplementary Table 5. The matrix of inner product of hub genes with MAPT in control group.

|  | **LCK** | **ZAP70** | **CD44** | **MAPT** |
| --- | --- | --- | --- | --- |
| **LCK** | 39.89356353 | 10.67084261 | 0.014896535 | 0.440034135 |
| **ZAP70** | 10.67084261 | 40.72583844 | 0.010609914 | 2.440907998 |
| **CD44** | 0.014896535 | 0.010609914 | 8.25272277 | 0.013673914 |
| **MAPT** | 0.440034135 | 2.440907998 | 0.013673914 | 5.141352162 |

**Supplementary Table 6.** The matrix of inner product of hub genes with MAPT in AD group.

|  | **LCK** | **ZAP70** | **CD44** | **MAPT** |
| --- | --- | --- | --- | --- |
| **LCK** | 5.727069923 | 0.319953916 | 6.273151048 | 1.335080462 |
| **ZAP70** | 0.319953916 | 0.737007391 | -0.011864764 | 0.749441449 |
| **CD44** | 6.273151048 | -0.011864764 | 7.566364871 | 2.774457681 |
| **MAPT** | 1.335080462 | 0.749441449 | 2.774457681 | 7.187986299 |
